# Liver Failure, Renal Failure, and a Rash: An Unusually Severe Case of Multi-Organ Failure Due to Murine Typhus

**DOI:** 10.7759/cureus.16661

**Published:** 2021-07-27

**Authors:** Stephanie Wachs, Brandon Kuiper

**Affiliations:** 1 Internal Medicine, Brooke Army Medical Center, Fort Sam Houston, USA; 2 Gastroenterology, Brooke Army Medical Center, Fort Sam Houston, USA

**Keywords:** murine typhus, rickettsia typhi, liver failure, renal failure, rash, vector-borne illness

## Abstract

Murine typhus is a vector-borne disease transmitted to humans via fleas and typically causes an infection hallmarked by nonspecific, mild symptoms of fever, rash, and headache. More severe diseases, while rare, can occur. We present a complicated case of murine typhus resulting in liver and renal failure.

Our patient was a healthy 64-year-old Hispanic gentleman who presented to his local emergency department (ED) in Southwest Texas for fevers, chills, and myalgia progressing to dyspnea, fatigue, and jaundice. He was transferred to a Central Texas transplant hospital given concern for impending liver failure as well as acute kidney injury later requiring hemodialysis. Broad-spectrum antibiotics were narrowed to empiric doxycycline with eventual improvement in his lab values and symptoms. The return of *Rickettsia typhi* antibody lab values later revealed murine typhus to be the cause of his disease.

## Introduction

This case was previously presented as a virtual presentation at the 2020 TriService ACP Conference on September 11, 2020, the 2020 South Texas ACP Conference on October 1, 2020, and the 2020 Texas ACP Conference on November 7, 2020. It was also presented as an abstract and poster in the Resident/Fellow Abstract Competition and ePoster competition, respectively, as part of the National ACP Conference on April 29, 2021.

Murine typhus, a disease caused by *Rickettsia typhi* and transmitted via fleas that reside on vectors such as rats, cats, and opossums occurs worldwide, though in the United States, prevalence is highest in Texas and California [1]. The disease is often self-limited and typically presents with nonspecific symptoms of fever, rash, and headache, though more severe disease can rarely occur [2]. In Texas, annual reported cases have steadily increased from 157 reported in 2008 to 738 in 2018 with geographic areas of incidence expanding as well [3]. It is unclear why incidence continues to rise, though theories involving climate change, migration, and possible re-establishment in reservoir populations have been proposed [4]. Given the potential seriousness of this infectious disease and its increasing incidence, it is essential that physicians in Texas and the Southwestern United States be well versed in both typical and atypical presentations of murine typhus.

## Case presentation

A healthy 64-year-old Hispanic gentleman from South Texas without significant past medical history presented several times to his local ED with non-specific cold sweats and chills progressing to diffuse myalgia. After nearly a week of worsening severity, he presented to his local ED and tested negative for coronavirus disease 2019 (COVID-19), influenza, and pneumonia and was sent home with directions for conservative care. Over the next week, he then developed shortness of breath, fatigue, and darkening of his urine that led him to the ED once more where he was COVID-19 negative a second time and was again sent home. Thirteen days after the start of his illness, he again presented to the ED at the behest of his partner who noticed jaundice and scleral icterus; she was worried he was dehydrated and listless now with a fever of 102 degrees Fahrenheit. He was admitted for acute kidney injury and possible sepsis and was started on broad-spectrum antibiotics with Vancomycin and Cefepime. He remained in the local hospital for less than 48 h when his lab work showed rising liver-associated enzymes {aspartate transaminase [AST] of 265 U/L (normal range: 13-40 U/L), alanine aminotransferase (ALT) of 184 U/L (normal range: 9-51 U/L), hyperbilirubinemia, and thrombocytopenia (platelet count of 54,000 per mm3; normal range: 150,000-400,000 per mm3)]} concerning for impending acute liver failure without clear etiology. He was then transferred to the ICU of a transplant center in Central Texas in light of the concern for developing liver failure with a Model for End-Stage Liver Disease (MELD) score of 32 (portending a 52.6% three-month mortality) and worsening renal injury (creatinine 1.5 mg/dL, normal range: 0.60-1.25 mg/dL).

**Figure 1:**
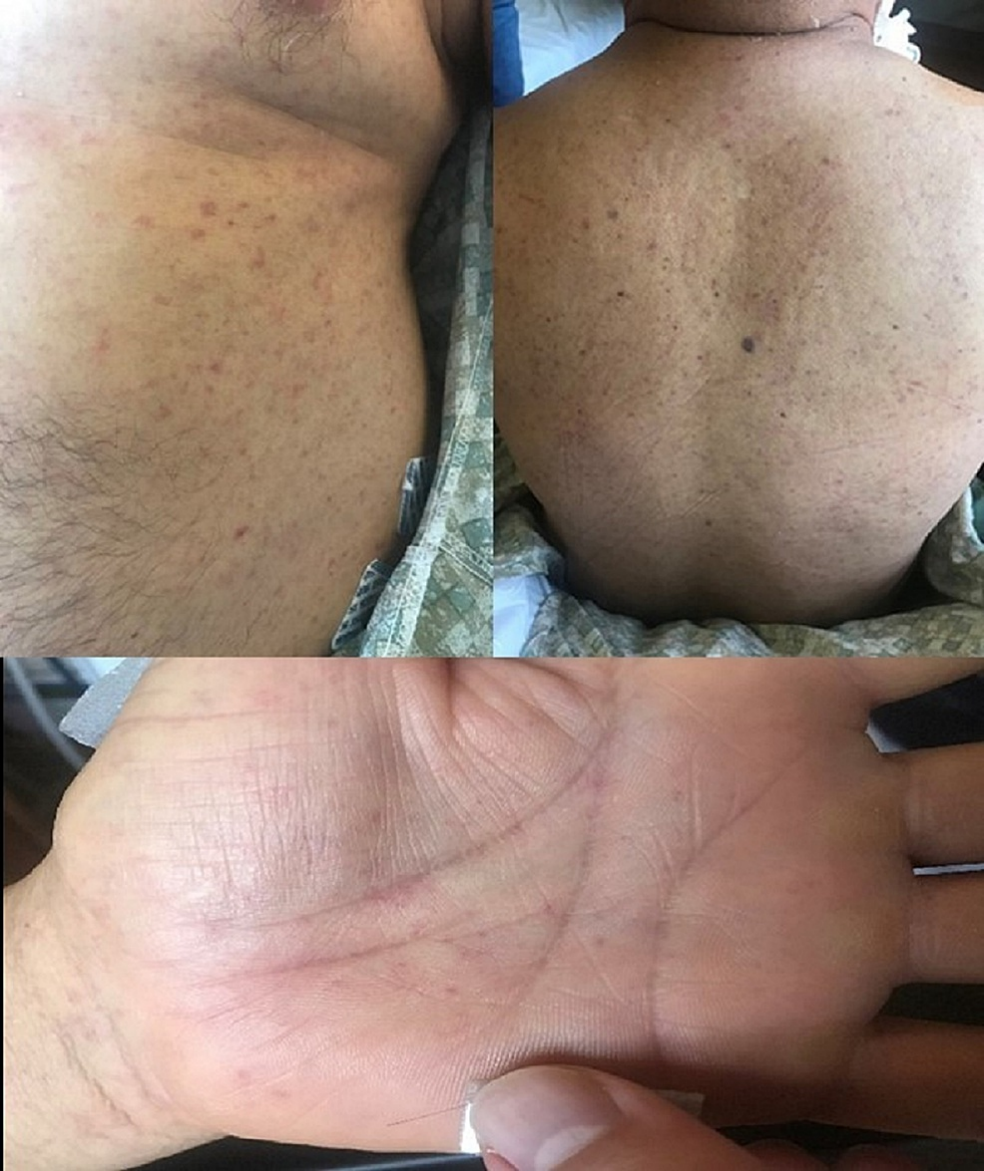
A 64-year-old man with life-threatening infection and multi-organ failure due to murine typhus. Examination was notable for a diffuse, erythematous maculopapular rash affecting the chest, abdomen, back, and palms.

On arrival, approximately 14 days after onset of his first symptoms, it was noted that he had subsequently developed a diffuse maculopapular rash over his chest, abdomen, back, and upper and lower extremities including the palms of his hands (Figure 1). It was neither pruritic nor painful and did not seem to coalesce. It was unclear when and where the rash originally started and in what pattern it spread. In light of a new febrile rash with both renal and hepatic failure, the differential was wide and included autoimmune, vasculitic, and infectious etiologies such as: systemic lupus erythematosus, microscopic polyangiitis, granulomatosis with polyangiitis, polyarteritis nodosa, sjogrens, sarcoidosis, erythema multiforme, mycosis fungoides, cryoglobulinemia, drug eruptions, secondary syphilis, human immunodeficiency virus, viral hepatitis B and C, Epstein-Barr Virus, cytomegalovirus, parvovirus B19, Rocky Mountain Spotted Fever, murine typhus, typhoid fever, bartonella, toxoplasmosis, coxsackie, measles, reactive arthritis, leptospirosis, and malaria. The following tests were ordered on admission: anti-nuclear antibody, rheumatoid factor, double-stranded DNA antibody, RNP antibody, SCL-70 antibody, SS-A antibody, SS-B antibody, centromere antibody, complement levels, glomerular basement membrane antibody, C-ANCA, P-ANCA, syphilis antibody, hepatitis A antibody, acute and chronic hepatitis B antigen and antibodies, hepatitis C antibody, acute and chronic Rocky Mountain Spotted Fever Rickettsial antibodies, acute and chronic *Rickettsia typhi* antibodies, Bartonella henselae antibodies, and Bartonella quintana antibodies.

**Figure 2:**
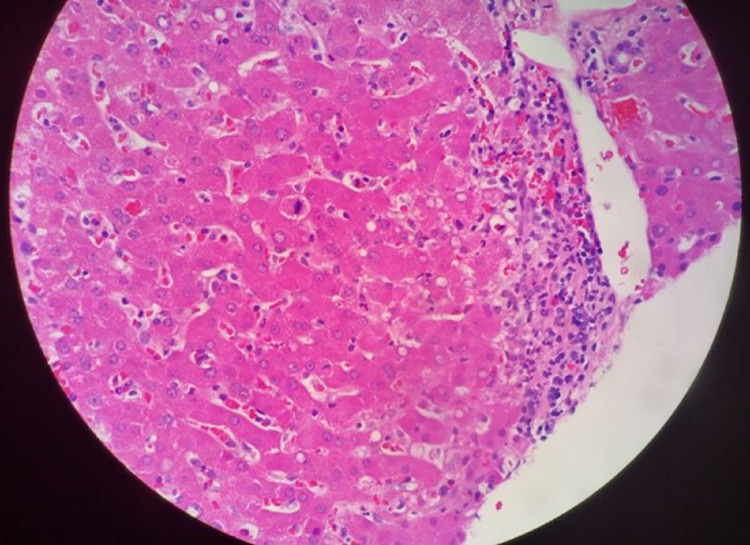
Liver biopsy demonstrated neither significant steatosis nor significant fibrosis but did feature prominent neutrophilic infiltration consistent with systemic inflammation attributed to murine typhus infection.

On arrival to the transplant hospital, laboratory markers were remarkable for AST of 302 U/L , ALT of 152 U/L , alkaline phosphatase (ALP) of 217 U/L (normal range: 34-122 U/L), total bilirubin of 7.8 mg/dL (normal range: 0.2-1.2 mg/dL), albumin of 1.8 g/dL (normal range: 3.4-5.4 g/dL), international normalized ratio (INR) of 1.4 (normal range: 0-1.1), platelet count of 35,000 per mm3, C-reactive protein (CRP) of 129 mg/dL (normal range: 0-0.49 mg/dL), and procalcitonin of 38.8 ng/mL (>9.99 ng/mL suggests a severe systemic inflammatory response). The patient and his partner confirmed that he had no known prior history of renal or liver disease and was absolutely abstinent from alcohol use. In fact, he had just months before undergone a routine physical exam and was found to be in good health. Liver ultrasound done on admission, however, was suggestive of cirrhotic morphology. To definitively evaluate this, he underwent a liver biopsy on hospital day 3 which showed no evidence of fibrosis (making cirrhosis unlikely), <5% fatty infiltration (ruling out hepatic steatosis), and prominent deposition of neutrophils (Figure 2) suggestive of an inflammatory response to systemic infection. Further studies were unremarkable and included a viral hepatitis panel, phosphatidylethanol (PETH) level, and evaluation of autoantibodies to detect autoimmune hepatitis and other underlying liver diseases (anti-mitochondrial antibody, anti-smooth muscle antibody, anti-nuclear antibody, ceruloplasmin, and alpha 1 antitrypsin). It was concluded that his liver injury was most likely secondary to an infection, though the offending organism was not yet identified. His coagulopathy and thrombocytopenia were managed supportively with standard transfusion thresholds.

**Figure 3:**
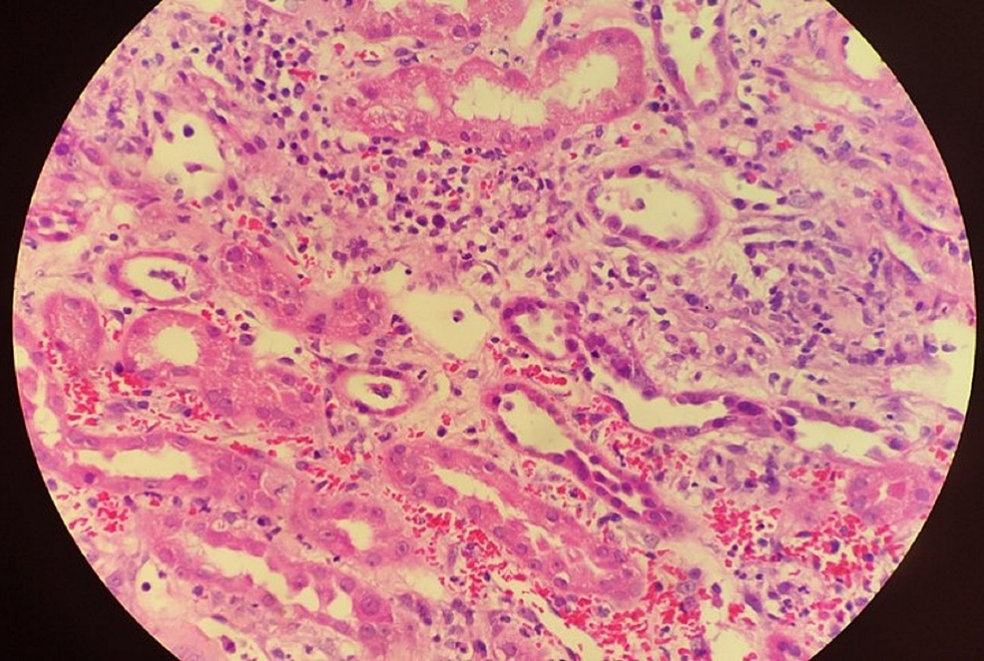
Renal biopsy demonstrated acute tubulointerstitial nephritis with fibrin thrombi in the venules, interstitial fibrosis, and tubular atrophy, which are consistent with overlap of AIN and ATN. AIN, acute interstitial nephritis; ATN, acute tubular necrosis

**Figure 4:**
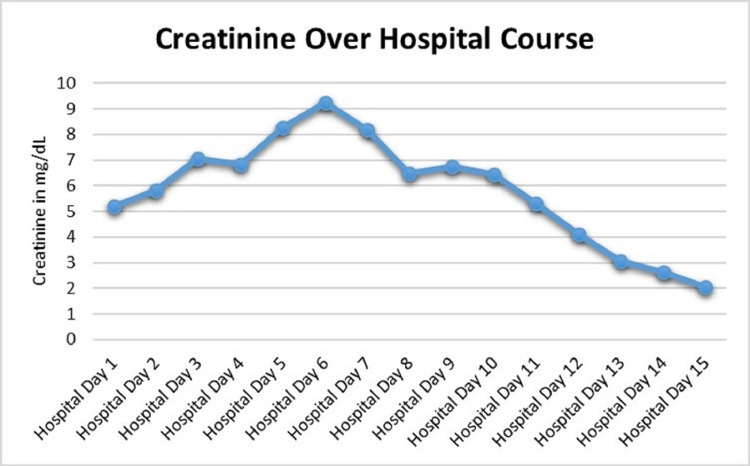
Renal function, as estimated by creatinine, trend over hospital course. Creatinine peaked on hospital day six with subsequent hemodialysis on days six to seven with steroids (solumedrol) to treat AIN on days seven to eight. AIN, acute interstitial nephritis

Renal ultrasound was completed shortly after arrival to the transplant hospital to investigate the patient’s acute kidney injury (creatinine on arrival of 5.21 mg/dL, normal range: 0.60-1.25 mg/dL). The ultrasound was unremarkable for abnormalities in renal size, morphology, or flow. A renal biopsy was performed three days thereafter demonstrating acute tubulointerstitial nephritis with fibrin thrombi in the venules, interstitial fibrosis, and tubular atrophy (Figure 3). These findings were unusual in that they demonstrated simultaneous acute tubular necrosis (ATN) and acute interstitial nephritis (AIN). He was given high-dose steroid therapy with 500 mg of IV solumedrol daily for three days for treatment of AIN on hospital days six through eight. Persistent elevations in creatinine (creatinine peaked to 9.24 mg/dL), electrolyte abnormalities, metabolic acidosis, and altered mental status, in the context of severe uremia, necessitated hemodialysis on hospital days six and seven. His renal function eventually recovered, and he did not require long-term dialysis (Figure 4).

**Figure 5:**
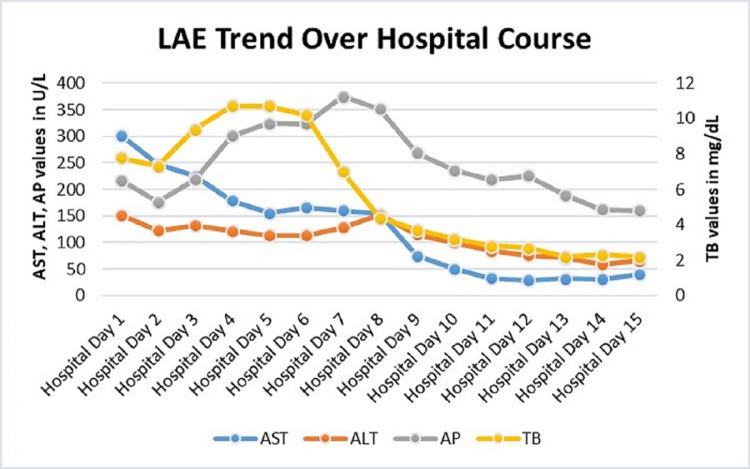
Trend of LAE over hospital course. LAE, liver-associated enzymes; AST, aspartate aminotransferase; ALT, alanine aminotransferase; TB, total bilirubin

In light of a wide differential for multi-organ failure and rash that included vasculitic disease, autoimmune conditions, and atypical rickettsial infections, he was started on empiric Doxycycline on hospital day two. On hospital day three, broad-spectrum antibiotics were stopped due to negative urine and blood cultures from the local hospital and the transplant hospital. Consultants during his hospital stay included hepatology, nephrology, and infectious disease with treatments as aforementioned. Over his 15-day hospital course, his symptoms, fever, and clinical markers all improved (Figure 5). Though empiric treatment for murine typhus with Doxycycline was started early in his hospital course, it was not until hospital day 15 that the diagnosis was confirmed with the return of *Rickettsia typhi* IgG of 1:256 and IgM of 1:128. Pointed questioning later revealed that he had recently taken in a stray kitten prior to his illness, which we suspect is the source of flea-borne transmission.

## Discussion

This case illustrates rarely seen severe manifestations of an often-overlooked vector-borne infectious disease. Murine typhus can present with nonspecific symptoms and mimic other disease processes, making lab-confirmed diagnosis difficult without a high index of suspicion. The most common symptoms caused by murine typhus are fever, headache, rash, and arthralgia. While elevated liver-associated enzymes and renal dysfunction are reported, a severe case of multi-organ failure requiring dialysis, as in this case, is exceedingly rare.

Hepatic dysfunction is a relatively common sequelae of murine typhus, as it is with other mild systemic infections. Laboratory abnormalities are common and include elevations in liver-associated enzymes (AST and ALT), hypoalbuminemia due to inflammation, and thrombocytopenia due to sequestration. In a retrospective case series of fatal flea-borne typhus cases in Texas from 1985 to 2015, the most common laboratory abnormalities were thrombocytopenia (82%) and elevated hepatic transaminase levels (64%) [5]. Similarly, in 90 cases of murine typhus in Texas from 2016 to 2019, 94% of cases had an AST >50, and 83% had an ALT >50 [6]. However, coagulopathy is rarely reported and cirrhotic morphology on imaging has not been described, which makes this a particularly peculiar aspect of this presentation. Due to the relatively small sample of the liver parenchyma obtained with biopsy, it is possible to miss patchy evidence of steatosis or fibrosis, and we cannot rule out the presence of previously undiagnosed non-alcoholic fatty liver disease (NAFLD). As seen in presentations of other acute and chronic liver diseases, overlap with fatty liver often accentuates the severity of the presentation in a second-hit manner; this could possibly explain his more severe symptoms. As the prevalence of NAFLD increases in the United States, providers may encounter traditionally minor hepatic insults with more severe manifestations in the future. 

Acute kidney injury is a common manifestation of murine typhus. In a systematic review of 33 case series from 1985 to 2016, 53 out of 1756 patients had an acute kidney injury with no mention of any of these necessitating hemodialysis [7]. Renal dysfunction is typically caused by a pre-renal lack of perfusion, as it is in most cases of infection-related acute kidney injury. Similar to this case, there are some reports of AIN due to murine typhus [8]. In our patient, we believe that his severe disease manifested as sepsis resulting in acute tubular and interstitial necrosis leading to profound renal failure. Another consideration violating Occam’s razor could be ATN related to sepsis with the addition of AIN as a drug side effect from the preceding antibiotics. Murine typhus is not known to directly damage the renal parenchyma or tubular system.

As aforementioned, rash is a frequent presenting symptom of murine typhus. Most commonly, the rash is macular or maculopapular and located on the trunk, mostly sparing palms and soles [7]. Our patient’s rash involved nearly the entire body including the trunk, back, legs, arms, and palms, which is dissimilar from most reports of the distribution and progression of the rash associated with murine typhus. A drug reaction to antibiotics is another consideration that could explain the differences between the typical presentation and that of this patient. 

This case of murine typhus is unique in its severity with acute renal failure due to AIN and ATN requiring dialysis, as well as elevated liver-associated enzymes, elevated bilirubin, thrombocytopenia, and mild coagulopathy concerning liver failure. Furthermore, the marked elevations in C-reactive protein (CRP) of 129 mg/dL and procalcitonin of 38.8 ng/mL were consistent with severe infection related to murine typhus as prior estimates suggest only 14.2% of patients with infections from rickettsioses mount a procalcitonin >2.0 ng/mL [9]. Following our literature review, we feel this is one of the more extreme presentations of murine typhus described, as this is often a non-specific and self-limited disease that rarely requires hospitalization let alone ICU admission and hemodialysis. Additionally, this case adds to the literature a description of liver and kidney biopsy findings in the setting of acute murine typhus, which is often not obtained given the more typical self-limited nature of this disease in most instances. The patient was from a county in Southwest Texas which had only one case of murine typhus reported from 2008 to 2018. Due to its rarity, the index of suspicion was low, but it is still important to consider this diagnosis given the number of murine typhus cases have been rising in Texas and creeping into areas previously unaffected over the last 12 years [3]. Explanations for this change in prevalence and distribution include climate change and the possibility that the pathogen is becoming re-established in populations home to the reservoirs extending from the Rio Grande Valley where the pathogen has remained endemic, thus spreading more easily throughout the state [4]. 

## Conclusions

While a vague pattern of symptoms with fevers, chills, headaches, myalgia, and laboratory abnormalities, such as thrombocytopenia and elevated liver-associated enzymes, may have a very broad differential, we posit that murine typhus must be on the list in light of the changing nature of this disease. Recognition that murine typhus can actually cause severe illness with multi-organ failure, as in the patient presented, is critical to initiating early appropriate therapy in order to mitigate severe and even fatal systemic consequences in affected patients. As murine typhus becomes more prevalent in Texas and the Southwest United States, physicians working in endemic areas should become familiar with its presentation and differentiating features and consider evaluation for this condition, especially in cases of unexplained rash with some degree of hepatic and renal dysfunction.
